# Supplementary material for: Indirect questioning methods for sensitive survey questions: Modelling criminal behaviours among a prison population
Source: PLoS One. 2021 Jan 22;16(1):e0245550. doi: 10.1371/journal.pone.0245550 (PMC7822331; doi:10.1371/journal.pone.0245550)
Supplement: Attachment — Submitted filename: reply_referees.docx [file pone.0245550.s001.docx]

Replies to Comments by Reviewer 1

Dear Sir/Madam

We are very grateful for your valuable comments and suggestions regarding our paper and have taken them into consideration in preparing this revised version, which we hope will be considered satisfactory.

A point-by-point replies to the issue you had raised follow.

1. It shows that the randomized response technique does what it always does, revealing a higher frequency of admitting having done various crimes than direct questioning does. The article does not give us a reason why we should have doubted this outcome for this special case (prison inmates, a number of crimes that according to the authors have not yet been researched). So what is new here? It is completely in line with eg the meta-analysis of Lensvelt et al.

*The novelty of this work with respect to other RRT works lies in the investigated population: the prison population. The social desirability bias is expected to be observed in people who follow the rules and comply with the laws, but in prison populations, where criminal behaviour is the order of the day, and some are very frequent (such as drug use) has not been investigated and it could be questioned whether, despite being very common behaviours, this population has problems in openly acknowledging its practice.*

*The RR technique was developed in an attempt to improve the quality of self-reported survey research, but it is not very often applied in prison population contex.*

2. Why should we be interested in criminal prevalence for theft, violence against property, reckless driving, arson in a population incarcerated for theft, violence, sex crimes, homicide murder, “other”?

*Indeed, this point is not one of the essential objectives of the work, and we have modified the research questions, focusing on the main questions, the social desirability bias among prison population.*

a. The article does not analyse the special subgroups that are incarcerated for theft or violence, which may be interesting as we know the true behaviour (if we trust the Spanisgh judges).

*We have included a couple of graphs that show the prevalence of the sensitive variables theft and violence for the type of crime committed, observing that the direct prevalence of theft by people who have been accused of theft is 0.8333. If we observe this value in the indirect case, it reaches a prevalence of 1, this difference being statistically significant.If we focus on the violence variable, in the direct case we obtain a prevalence of 0.2857, while in the indirect case it reaches 0.5595, but this difference is not significant.In this case, we have obtained a low prevalence value, since the sensitive variable refers to violence towards objects of value and the crime committed is referred to general violence*

*Comments have not been included in the article for not being relevant and for not increasing the length of the article following your suggestions*

b. It is not true that no studies have been done on RR for arson (Durham & Lichtenstein) or theft (egWimbush& Dalton).

*Thank you for your comment. Indeed, the indicated works have considered these issues, although the populations to be studied (personnel with access to cash or products easily converted to cash and undergraduate students in sociology courses) are very different from what we consider. The paragraph has been changed, including these works and emphasizing the novelty regarding the population considered.*

3. The authors tell us that no studies have been done on the relation crime – impulsivity. That is completely wrong, indeed it is a very active field in criminology, following Wilson &Hernstein; Gottfredson).

*Thank you for your comment. Indeed, we are studying impulsivity and compulsivity. That is, impulsivity and compulsivity characterize a wide range of disorders, and, in some cases, they appear to overlap; for instance, many disorders can be characterized by both impulsivity and compulsivity, either simultaneously or at different times (Grant &Chamberlain, 2019). Impulsive behaviours can often be controlled, while compulsive behaviours may require more specialized and multifactorial (biological, psychological, and social) interventions, as they are often part of a more serious problem. Impulsivity and compulsivity are traits that are thought to underlie violent behaviour (Fitzpatrick, Dworkin, Zimmerman, Javorka&Kaysen, 2020; Olver, Kingston &Sowden, 2020).*

*The behaviour of impulsive patients aims to alleviate anxiety or discomfort and to satisfy the desire for pleasure, excitement, or gratification. Patients at the end of the compulsive spectrum have an exaggerated sense of threat from the outside world and perform rituals/routines such as obsessive-compulsive behaviours in order to neutralize the threat or reduce harm. This latter point marks compulsive or risk-averse behaviours that are characterized by an overestimation of the probability of future harm. Thus, some compulsive patients engage in behaviours or rituals to achieve short-term benefits (stress relief) in spite of the long-term negative consequences (Figee et al., 2016; Hollander et al., 2016). The ground-breaking nature of this study is reflected in this is the first time that the construct of compulsivity will be defined in prison populations and delimited differences among impulsivity and compulsivity. This study is the first time that impulsivity and/or compulsivity in men who commit violent crimes.*

4. While logistic regression results are given, we do not get much interpretation out of them.
a. Should you not compare and comment on the size of the various coefficients compared over tables (eg what to make out of the result that “married” is significant in table 3 and insignificant in all other tables?).
b. What about effect sizes? Is “obsessive/compulsive” an important factor, with exp(estimates) of 1.04 to 1.07? Oristhisnegligible.

c. You should comment on an estimate of 13731595.8244. It points to such a skew distribution of the dependent that the logistic regression is probably numerically instable. (Figure 44 shows us that indeed a 100% prevalence in some categories, which is a problem for the algorithm).

*To interpret the magnitude of the effect sizes, Table 2 includes the Cohen's delta, considering as a group the method (direct, indirect) used.*

*The regression section has been changed following the instructions of the second referee.*

*We faced several problems in estimating the regressionmodels, probably due to the relatively small number of casesin our study and the low statistical efficiency of RR data. Some models did not convergeand the results were unstable.*

*Therefore, we present a logisticmodel only for the variables theft, drugs and arsonsince results from different estimation techniques were stablefor thesevariables.*

*Problems and inconsistent results using multivariate regressionwith indirect questioning techniques have also been reported by Comça and Postelnicu (2013) and Wolter and Laier (2014). Our conclusion regarding the difficulties that we and other authors have encountered is thatregression analysis using RRT data is not (yet) as straightforwardas sometimes claimed. Different estimators yieldunstable results and large sample sizes are necessary.*

*On the other hand, some of the logistic regression developments have not yet been developed for the randomized response case, so we find these limitations*

5. The paper is unduly long.

*We have tried to decrease the length of the article, we have eliminated some formulas, some figures have been moved to the appendix and we have eliminated the regression models for some variables, focusing only on those in which the logistic regression is numerically stable.*

Thanks again for your comments and time.

The authors.

References

Comça, M. &Postelnicu, C. (2013).Measuring social desirabilityeffects on self-reported turnout using the item count technique.International Journal of Public OpinionResearch, 25(2), 153 172.

Wolter, F., &Laier, B. (2014).The Effectiveness of the Item Count Technique in Eliciting Valid Answers to Sensitive Questions.An Evaluation in the Context of Self-Reported Delinquency.*Survey Research Methods*, *8*(3), 153-168. https://doi.org/10.18148/srm/2014.v8i3.5819

Figee, M., Pattij, T., Willuhn, I., Luigjes, J., van, d. B., Goudriaan, A., . . . Denys, D. (2016). Compulsivity in obsessive–compulsive disorder and addictions. European Neuropsychopharmacology, 26(5), 856-868. doi:http://dx.doi.org/10.1016/j.euroneuro.2015.12.003

Fitzpatrick, S., Dworkin, E. R., Zimmerman, L., Javorka, M., &Kaysen, D. (2020). Stressors and drinking in sexual minority women: The mediating role of emotion dysregulation. Psychology of Sexual Orientation and Gender Diversity, 7(1), 46-54. doi:http://dx.doi.org/10.1037/sgd0000351.

Grant, J. E., & Chamberlain, S. R. (2019). Obsessive compulsive personality traits: Understanding the chain of pathogenesis from health to disease. Journal of Psychiatric Research, 116, 69-73. doi:http://dx.doi.org/10.1016/j.jpsychires.2019.06.003.

Hollander, E., Doernberg, E., Shavitt, R., Waterman, R.J., Soreni, N., Veltman, D.J., Sahakian, B.J. y Fineberg, N.A. (2016). The cost and impact of compulsivity: A research perspective. European Neuropsychopharmacology, 26(5),800-809. doi:http://dx doi: 10.1016/j.euroneuro.2016.02.006.

Olver, M. E., Kingston, D. A., &Sowden, J. N. (2020). An examination of latent constructs of dynamic sexual violence risk and need as a function of indigenous and nonindigenous ancestry. Psychological Services, doi:http://dx.doi.org/10.1037/ser0000414.

Replies to Comments by Reviewer 2

Dear Sir/Madam

We are very grateful for your valuable comments and suggestions regarding our paper and have taken them into consideration in preparing this revised version, which we hope will be considered satisfactory.

We have also taken into account the comments of the other referee and following his instructions, we have tried to decrease the length of the article: we have eliminated some formulas, some figures have been moved to the appendix and we have eliminated the regression models for some variables, focusing only on those in which the logistic regression is numerically stable.

A point-by-point replies to the issue you had raised follow.

1.-The introduction is clear and manages to cover the topics studied in this manuscript.

*Thank you for your comment.*

2.-Please move the participants’ information from page 9 (table 1) to the methods section, so the reader can have a better reading of the methods and results sessions.

*Table 1 has been moved to the methods section.*

3.-Please add more information related to the construction of the survey. For example, was it online? Was any software used for the design? Was itself-administered?

*All the measures were completed face to face. As you should read in the article, this study was completed sociodemographic information regarding the crime for which the prison sentence according to applicable legislation by Eva Castillo Fernandez (researcher of this study).*

4.-Within the inclusion criteria, it was taken into account the general cognitive state?

*The inclusion criteria were having between 18 and 55 years old. Participants were excluded according to the following exclusion criteria: being older than 55 years, with physical, psychiatric illness (schizophrenia or depression), and are currently undergoing psychopharmacological treatment.*

*Procedure: Potential participants were interviewed individually to check whether they met the inclusion criteria and were offered the opportunity to participate in the research. Afterward, they have completed the Symptom Inventory (SCL-90-R). The participants then underwent an individual session in which they were complete the measures described below. At the beginning of the session, the participants were reminded that they had the right to abandon the study at any moment and were sign a written informed consent form if they agree to participate. At the end of the session, participants were debriefed and thanked for their participation.*

5.-In relation to the participants who had no schooling (n = 125), how did they answer the test? Was there a way to ensure they understood the survey questions?

*All the participants could read and write. During the study measurements, the researchers answered the questions that the participants had.*

6.-My main concern is related to Research Question 3. Tables 3-7 show the regression coefficients (p’s <0.05). However, they do not show the final model quality and its ability to predict / infer. My suggestion is using some cross-validation techniques, such as k fold validation or other techniques. Also, the authors can use statistical learning strategies (e.g. OneR, SimpleCart, and REPTree algorithms) for feature selection to determine the best variables for classification and compared models fits using the classification metrics: True Positive Rate (TPR), False Positive Rate (FPR), Precision and Accuracy, etc.; and then use cross-validation technical. The model presented in the manuscript could have an overfitting effect and not predict in a new sample / population.

*The regression section has been changed trying to respond to the indications of both referees.*

*In each of the regression models, we have included the value of the Pearson statistic as a measure of goodness of fit, which has been calculated with GLMMRR package of the R software.We have not included classification metrics since the objective in RRT is not to predict specific values (difficult to do given the randomness of the response mechanism for each individual), but to estimate proportions or means. This is in line with other logistic regression work in RRT such as Van der Hout et al (2007),Cruyffet al (2016), Fox et al (2019).*

*The measures and techniques to which it refers have not yet been developed in the case of randomized response. To date, we do not know of any work that uses these techniques with RR data and it is one of the objectives we have in our future research.*

*On the other hand we faced several problems in estimating the regression models, probably due to the relatively small number of cases in our study and the low statistical efficiency of RR data. Some models did not converge and the results were unstable.*

*Therefore, we present a logistic model only for the variables theft, drugs and arson since results from different estimation techniques were stable for these variables.*

*Problems and inconsistent results using multivariate regression with indirect questioning techniques have also been reported by Comça and Postelnicu (2013) and Wolter and Laier (2014). Our conclusion regarding the difficulties that we and other authors have encountered is that regression analysis using RRT data is not (yet) as straightforward as sometimes claimed. Different estimators yield unstable results and large sample sizes are necessary.*

7.-Please clarify the alpha value used for the analyzes performed. WasBonferronicorrectionused?

*The GLMMRR package internally uses the glm function of the stats package and from what we have seen in the help and we have found on the web it seems that multiple comparisons of any kind are not performed*

*Minor comments.

Please select the most representative graphics, I don't find very didactic to leave 15 similar graphics in a single manuscript.

*The new version includes 3 graphics and the rest have been moved to the appendix.*

Tables 3-7, please use scientific notation or use the expression p <0.001, for the p value = 0.000

*Done*

Thanks again for your comments and time.

The authors.

References

Van den Hout, Ardo, van der Heijden, Peter G.M., Gilchrist, Robert. (2007) The logistic regression model with response variables subject to randomized response.Computational Statistics & Data Analysis.Elsevier, vol. 51(12), pages 6060-6069.

Cruyff, M. J. L. F., Böckenholt, U., van der Heijden, P. G. M., & Frank, L. E. (2016). A Review of Regression Procedures for Randomized Response Data, Including Univariate and Multivariate Logistic Regression, the Proportional Odds Model and Item Response Model, and Self-Protective Responses. In A. Chaudhuri, T. C. Christofides, & C. R. Rao (Eds.), Data Gathering, Analysis and Protection of Privacy Through Randomized Response Techniques: Qualitative and Quantitative Human Traits, 2016 (pp. 287-315). (Handbook of Statistics; Vol. 34).Elsevier. https://doi.org/10.1016/bs.host.2016.01.016

Fox JP, Veen D, Klotzke K. Generalized linear mixed models for randomized responses. Methodology.2019; 15(1), 1-18. [doi:10.1027/1614-2241/a000153](https://doi.org/10.1027/1614-2241/a000153).

Comça, M. &Postelnicu, C. (2013).Measuring social desirabilityeffects on self-reported turnout using the item count technique.International Journal of Public OpinionResearch, 25(2), 153 172.

Wolter, F., &Laier, B. (2014). The Effectiveness of the Item Count Technique in Eliciting Valid Answers to Sensitive Questions. An Evaluation in the Context of Self-Reported Delinquency. *Survey Research Methods*, *8*(3), 153-168. https://doi.org/10.18148/srm/2014.v8i3.5819
